# Vocal smile is recognized but not embodied in autistic adults

**DOI:** 10.1016/j.isci.2025.113858

**Published:** 2025-10-25

**Authors:** Annabelle Merchie, Zoé Ranty, Claire Wardak, Nadia Aguillon-Hernandez, Frédérique Bonnet-Brilhault, Emmanuelle Houy-Durand, Jean-Julien Aucouturier, Marie Gomot

**Affiliations:** 1 ACTE at ULB Neuroscience Institute, Université Libre de Bruxelles, 50 Avenue F.D. Roosevelt, 1050 Brussels, Belgium; 2 Université de Tours, INSERM, Imaging Brain & Neuropsychiatry iBraiN U1253, 37032 Tours, France; 3 Excellence Center for Autism and Neurodevelopmental Disorders, EXAC·T, CHRU de Tours, Tours, France; 4 Centre Ressource Autisme Région Centre-Val de Loire, CHRU de Tours, Tours, France; 5 Université de Franche-Comté, SUPMICROTECH, CNRS, institut FEMTO-ST, Besançon, France; 6 STMS Lab IRCAM, CNRS, Sorbonne Université, Paris, France

**Keywords:** Physiology, Neuroscience, Behavioral neuroscience, Cognitive neuroscience, Psychology

## Abstract

Autism spectrum disorder (ASD) is frequently characterized by atypical responses to emotional prosody and a lack of response to social smiles. This study examined whether autistic adults show motor resonance to vocal smiles, as reflected in facial muscle activity, when listening to emotional vocal cues. Facial electromyography was recorded while autistic and neurotypical adults listened to sentences spoken with smiling or neutral prosody and judged their emotional content. Both groups accurately recognized smiling prosody, indicating intact perceptual abilities in autism. However, only neurotypical participants showed enhanced zygomaticus activation in response to smiling voices, whereas autistic participants did not modulate facial muscle activity despite correct recognition. This dissociation between identification and motor reactivity suggests that autistic individuals can accurately recognize vocal emotions, but these are not embodied. These findings provide insights into the mechanisms that shape social engagement in ASD and may inform therapeutic approaches targeting emotional embodiment.

## Highlights

• Autistic and neurotypical adults accurately recognized smiling vocal prosody
• Neurotypical adults showed specific motor reactivity to smiling voices
• Autistic adults did not display motor resonance for a vocal smile
• Vocal emotions are identified but not embodied in autistic adults

## Graphical abstract

**Figure:**
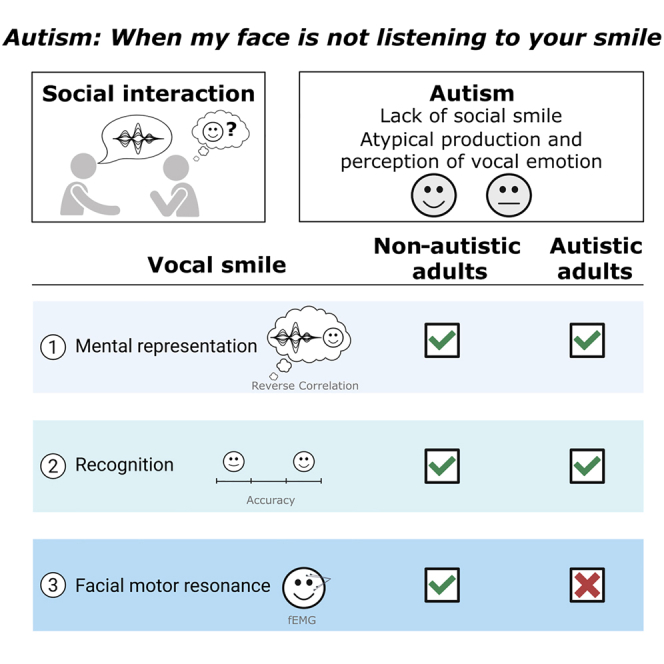

## Introduction

Atypical facial expressions, described as reduced or flat, have been reported in autism.^1^ Differences in the production and perception of facial expressions can hinder the ability to convey emotions and intentions, which is crucial for social interaction. As a result, autistic individuals may not show typical responses to emotional cues,^2^ leading to misunderstandings and the perception of being less engaged or empathetic.^3^ Similarly, their limited facial expressiveness can make it challenging to form social connections, as neurotypical individuals may find it more difficult to understand the emotional states of autistic people.^4^^,^^5^ Building on these challenges in facial expressiveness, individuals with autism may also experience difficulties in producing and processing emotional prosody and exhibit atypical responses to these vocal cues.

### Emotional prosody in autism

Atypicalities in the production and perception of emotional prosody have been extensively documented in autism, in both clinical reports and experimental studies. Prosody production is often described as monotonous or exaggerated in autism,^6^^,^^7^ to the point that in the Autism Diagnostic Observation Schedule (ADOS), atypical prosody is one of the hallmarks for diagnosis in the language and communication dimension.^8^ These atypical patterns can impact social interactions and the ability to convey or understand emotional nuances in speech. In experimental situations, difficulties in the recognition of emotional prosody have also been reported in both autistic children^9^ and adults.^10^ However, some studies have reported that prosody identification can be similar to that of neurotypicals,^11^^,^^12^ and others indicate it may simply be less accurate^13^^,^^14^ (but see Leung et al.^15^ for a comprehensive review on emotional prosody perception and recognition in autism).

Given that the recognition of emotional prosody in autism varies across studies, it could be hypothesized that perceptive representations of vocal emotion can differ across participants, as suggested for the representation of facial expression.^16^ However, in a recent study, we found that neurotypical individuals and most autistic participants shared a similar internal representation of vocal smiles.^17^

### Motor resonance in autism

In the visual modality, a lack of motor facial resonance has been observed in autistic individuals when responding to various social cues, such as yawns^18^^,^^19^^,^^20^ and facial expressions.^2^^,^^18^^,^^21^^,^^22^^,^^23^ While neurotypical individuals demonstrate yawning contagion in response to auditory stimuli,^18^^,^^24^ this phenomenon is absent in autistic children,^25^ suggesting that sensory modality does not influence behavioral contagion in either neurotypical or autistic individuals. However, Magnée et al. demonstrated that motor resonance was atypical for autistic adults in response to visual-only emotion, while no difference with the response of neurotypicals was observed for audiovisual emotional stimuli.^22^ This finding raises the question of the facilitating effect of prosodic content on motor resonance in autism, but also of the modality of presentation of an emotional stimulus on motor and emotional resonance.

Among social cues, smiling is recognized as one of the most effective triggers of emotional resonance.^26^ In individuals with autism, difficulties in tasks involving the observation^21^ and the production of social smiles^27^(i.e., facial or vocal smiles that are directed toward others and serve a communicative, affiliative function^28^) have been reported. Smiles are also identifiable in the auditory modality, characterized by a robust acoustic signature that is comparable between autistic and neurotypical adults.^17^^,^^29^ Recent studies have further shown that autistic individuals may also process other socially relevant vocal cues, such as laughter, differently from neurotypicals, both at the neural and behavioral levels.^30^^,^^31^ In neurotypical and congenitally blind individuals, motor resonance in response to vocal smiles has been observed, leading to congruent motor responses.^32^^,^^33^^,^^34^ Moreover, research on cross-modal processing in neurotypical adults suggests that emotional vocalizations can elicit facial muscle responses even in the absence of visual input, reflecting an integrated sensorimotor response of vocal emotion.^35^^,^^36^ However, to date, no studies have specifically investigated facial motor resonance in response to purely emotional prosodic cues, such as vocal smiles, in autism.

### The present study

Given these insights, the objective of the current study was to investigate facial motor resonance in response to vocal emotions in autistic adults. Specifically, we measured facial muscle (*Zygomaticus major* and *Corrugator supercilii*) responses to smiling and non-smiling filtered phrases using facial electromyography (fEMG) in both autistic and neurotypical adults. Sentences were filtered using the typical vocal smile model extracted from Ponsot et al.,^29^ resulting in slightly smiling or unsmiling utterances. As in the passive listening task, no motor resonance could be observed,^37^ participants were asked to judge the prosody of the spoken phrases. Before conducting the EMG measurements, we checked whether the perceptual representations of vocal smiles were similar between the groups. This step was essential to ensure that any observed differences in facial motor responses could be attributed to differences in motor resonance rather than to disparities in perception and judgment.

## Results

### Representation of vocal smile and accuracy

**Figure 1:**
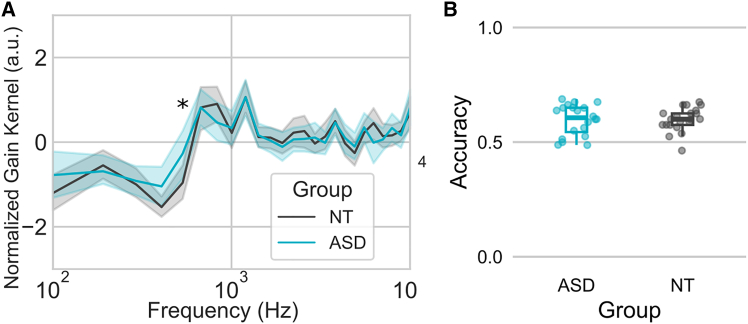
Representations of vocal smile from the reverse correlation task and accuracy in the EMG task (A) Averaged filter underlying the evaluation of vocal smile in ASD (blue) and NT (gray), as derived with reverse-correlation. Asterisks indicate significant differences between the two groups (two-tailed; paired sample t-tests; *p* < 0.05). (B) Accuracy in the EMG task in ASD (blue) and in NT (gray).

Representations of vocal smile were similar between groups with analogous acoustic frequency and amplitude features (Figure 1A). A statistical difference between groups was observed for only one frequency, 533 Hz (t(39) = 2.2; *p* < 0.05).

Perceptual accuracy was above the chance (0.5) level in both groups (NT t(20) = 8.83; *p* < 0.001; and ASD t(19) = 6.49; *p* < 0.001). There was no significant difference between the NT and ASD groups on the Accuracy at the EMG task (t(39) = −0.07, *p* > 0.9) (Figure 1B). The Bayesian t-test indicated moderate evidence in favor of the null hypothesis, suggesting no significant difference in perceptual accuracy between groups (BF_10_ = 0.294, error = 0.006%).

The correlation analysis revealed no significant correlation between the Kernel Distance and Perceptual accuracy when the NT and ASD groups were combined (r(39) = −0.67; *p* > 0.05). Additionally, no significant correlation was found within the individual groups, neither for the ASD group (r(18) = −1.08; *p* > 0.05), nor for the NT group (r(19) = 0.29; *p* > 0.05).

No significant correlations were identified between Perceptual accuracy and AQ (r(34) = 1.59; *p* > 0.05), nor EQ (r(34) = −0.70; *p* > 0.05).

### Motor resonance

#### Muscle activity

**Figure 2:**
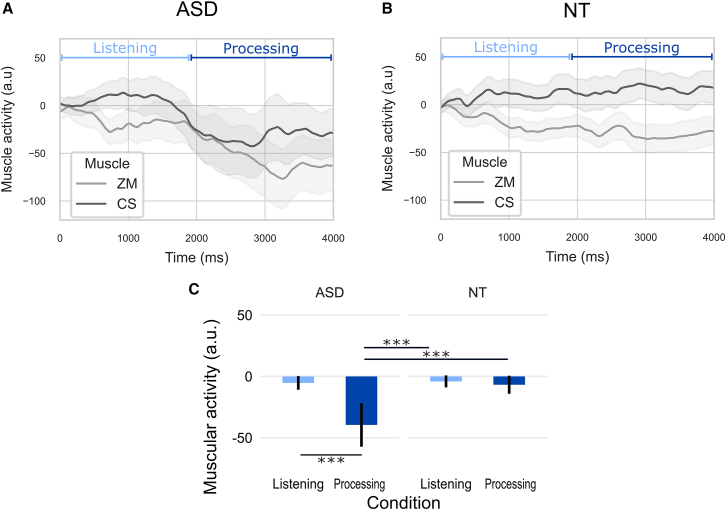
Temporal dynamics of facial muscle activity during sentence listening and processing sentences (A) Effect of Condition on Zygomaticus major (ZM) and Corrugator supercilii (CS) in ASD. (B) Effect of Timing on ZM and CS in NT. (C) Effect of Timing on muscular activity in ASD and NT – mean response amplitude in selected time windows: 0–1900 ms (Listening – light blue) and 1900–4000 ms (Processing – dark blue). Shaded areas and error bars represent the standard error of the mean.∗∗∗*p* < 0.001.

To evaluate the effect of Listening and Processing of sentences on muscular activity between Groups and within Muscles a repeated measures ANOVA was performed on mean muscular activity during Listening to sentences (0–1900 ms) and Processing (1900–4000 ms), referred to as Timing in the ANOVA. The ANOVA revealed a main effect of Muscle (F(1,39) = 9.44, *p* < 0.01, η2*p* = 0.07) with a larger contraction of the CS than ZM, and of Timing (F(1,39) = 5.70, *p* < 0.05, η2*p* = 0.02) (Figures 2A and 2B). These effects of Timing were specified by the significant interaction between Timing and Group (F(1,39) = 4.27, *p* < 0.05, η2*p* = 0.02) driven by the larger relaxation of muscles in the ASD group only during the processing of sentences (p_corr_ < 0.001) (Figure 2C).

As a main group difference was observed during the second time period (Processing phase), repeated measured ANOVAs were focused on a chosen 500 ms window (2900–3400 ms) during this phase in accordance with a previous study, which identified this time window as showing a significant increase in z-normalized fEMG activity in response to vocal smiles.^34^

An omnibus repeated measures ANOVA was conducted to analyze mean muscular activity, considering Group and Muscle as, respectively, between and within factors, in the 2900–3400 ms time window. Although muscular activity was smaller in ASD than in NT, no statistical difference was observed between groups (F(1,39) = 2.75, *p* = 0.11, η2*p* = 0.05). A significant effect of muscle was observed, indicating larger muscular strength for CS than for ZM muscles (F(1,39) = 11.95; *p* < 0.001; η2*p* = 0.09). Based on these results, the analysis was split by muscle to evaluate the effects of Filter and Choice. Group was used as a between-subject factor in the main ANOVAs, followed by separate analyses within each group to specify the effects of Filter and Choice independently.

#### Zygomaticus major activity

A repeated measures ANOVA was conducted to analyze the mean muscular activity of the ZM in the 2900–3400 ms time window, considering Group as between, and Filter and Choice as within factors. No main effects were found for any of the factors (*p* > 0.05) and no interaction, but a difference for Filter almost reached significance (F(1,39) = 3.95; *p* = 0.052; η2*p* = 0.01).

**Figure 3:**
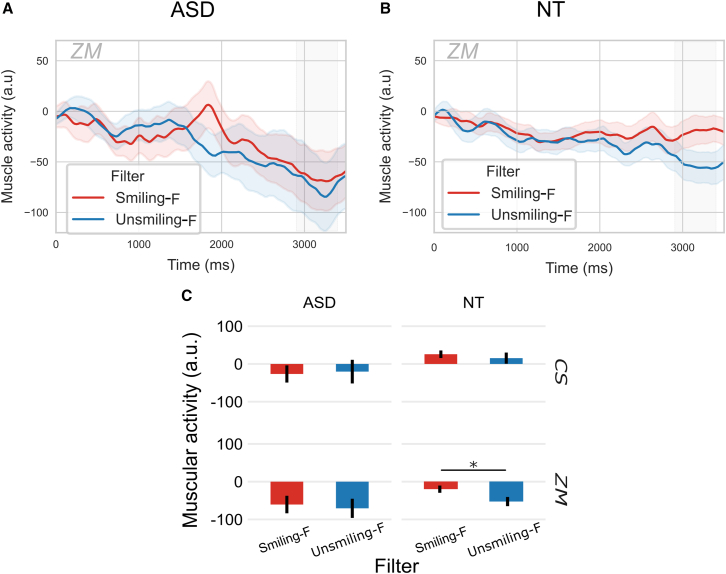
Modulation of facial muscle activity by acoustic filter (Smiling-F vs. Unsmiling-F) (A) Effect of Filter on ZM in ASD. (B) Effect of Filter on ZM in NT. (C) Effect of Filter on CS and ZM in ASD and NT – mean response amplitude in the window 2900-3400 ms (in gray in A and B time series). Shaded areas and error bars represent the standard error of the mean. ∗*p* < 0.05.

Considering the observed effect on time series (Figures 2A and 2B), the trend of the Filter effect and our previous study in NT showed an effect of Filter,^34^ even if no Group by Filter interaction was found significant, separate analyses were performed within each Group to specify these effects. These within-group analyses were exploratory and should be interpreted with caution, as they do not compensate for the lack of a significant interaction. In the ASD group, no difference was found for Filter (F(1,19) = 0.35; *p* = 0.56), choice (F(1,19) = 0.72; *p* = 0.41), or their interaction (F(1,19) = 0.41; *p* = 0.53) (Figures 3A–3C). In the NT group, a significant main effect of Filter was revealed (F(1,20) = 5.27; *p* = 0.03; η2*p* = 0.07), with greater activity for Smiling-F compared to Unsmiling-F filtered sentences (Figures 3B and 3C). No main effect of Choice (F(1,20) = 0.07; *p* = 0.79) or interaction between Filter and Choice (F(1,20) = 0.004; *p* = 0.95) was observed.

#### Corrugator supercilii activity

As for the ZM activity, a repeated measures ANOVA was conducted to analyze mean muscular activity of the CS in the 2900–3400 ms time window, considering group as between, and Filter and Choice as within factors. No main effects were found for any of the factors (*p* > 0.05), but significant interactions were observed between Group and Choice (F(1,39) = 4.73; *p* = 0.04; η2*p* = 0.01) and a tendency between Filter and Choice (F(1,39) = 3.68; *p* = 0.06; η2*p* = 0.008). Post-hoc pairwise comparisons revealed that the interaction between Group and Choice was due to the larger activity of the CS for Unsmile-C in NT that was different from Smile-C Choice in NT (p_corr_ < 0.05) and larger than the CS for Unsmile-C and Smile-C Choices in ASD (p_corr_ < 0.05). The interaction between Filter and Choice resulted from a statistical trend for the choice in Smiling-F filtered sentences, with greater activity in response to an Unsmile-C Choice (p_corr_ = 0.1), due to the larger activity in NT for Unsmile-C Choice.

**Figure 4:**
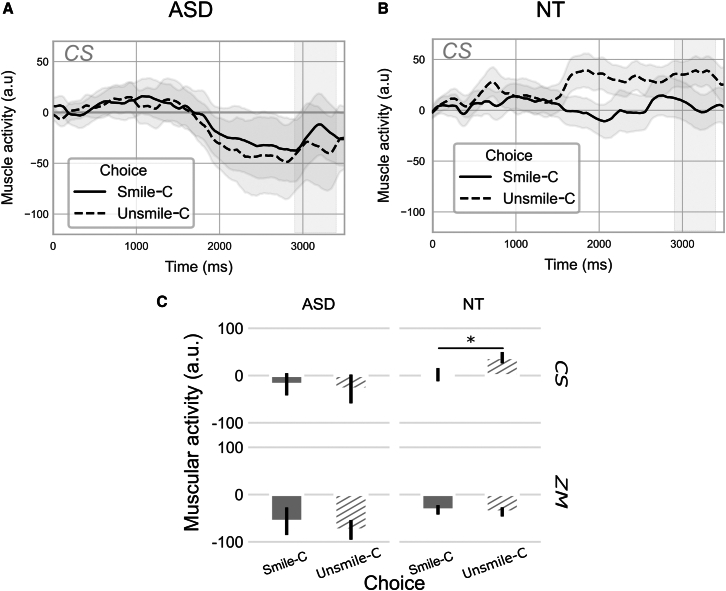
Facial muscle activity as a function of perceptual choice (Smile-C vs. Unsmile-C) (A) Effect of Choice on ZM in ASD. (B) Effect of Choice on ZM in NT. (C) Effect of Choice on CS and ZM in ASD and NT – mean response amplitude in the window 2900-3400 ms (in gray in A and B time series). Shaded areas and error bars represent the standard error of the mean. ∗*p* < 0.05.

Separate analyses within each group were performed to specify these effects. In the ASD group, no differences were found for Filter (F(1,19) = 0.14; *p* = 0.71), Choice (F(1,19) = 0.48; *p* = 0.50), nor their interaction (F(1,19) = 1.41; *p* = 0.25) (Figures 4A–4C). In the NT group, a significant main effect of Choice was revealed (F(1,20) = 5.60; *p* = 0.03; η2*p* = 0.05), with greater activity for sentences rated as Unsmile-C compared to Smile-C (Figures 4B and 4C). No main effect of Filter (F(1,20) = 1.40; *p* = 0.25) or interaction between Filter and Choice (F(1,20) = 3.84; *p* = 0.06) was observed.

### Correlation between measures

Previous analyses thus revealed that ZM activity was mainly modulated by filters, whereas the CS activity was sensitive to choice in NT. To simplify the expression of the effect of Filter and Choice on muscular activity, the difference between the filtered as Smiling-F and Unsmiling-F ZM response was computed as ZM_filter_, and the difference between the rated as Unsmile-C and Smile-C CS response was computed as CS_Choice_. Correlations between muscular activities were performed within each group. This analysis revealed no significant correlation between ZM_filter_ and CS_Choice_ in either the NT group (r(19) = 0.38; *p* = 0.71) or in the ASD group (r(18) = 0.64; *p* = 0.53). Indicating that the activation of one effector did not trigger a full congruent motor resonance.

## Discussion

The current study was the first to assess motor resonance to vocal emotion while controlling for perceptive representations of emotional prosody in a group of autistic adults. Differences in facial response to emotional prosody were observed with a large relaxation of both muscles (*Zygomaticus major* and *Corrugator supercilii*) during the processing of sentences in autistic adults. A total absence of motor resonance was observed in response to emotional prosody in a group of autistic adults, even if their representation of vocal smiles and their recognition of vocal emotion were as efficient as those of a group of neurotypicals. Even when vocal emotions were correctly perceived and judged, no motor resonance was observed in autistic adults. This contrasts with neurotypicals, for whom *Zygomaticus major* activity reflected the perceived emotion, while *Corrugator supercilii* activity was related to explicit emotional prosody processing. It should also be noted that, in neurotypicals, muscle relaxation is only observed in the corrugator supercilii in response to positive-valenced sounds. This finding is consistent with the literature on the visual modality.^38^^,^^39^^,^^40^ Most motor resonance studies have focused on early automatic responses typically observed within the first 500–1000 ms.^41^^,^^42^ In contrast, our extended analysis window allowed us to capture later stages of processing, which may reflect more complex or regulated components of motor resonance, such as top-down modulation or social disengagement^43^^,^^44^ in autistic and neurotypical adults.

The difference in overall muscular activity between autistic and neurotypical adults is notable. Distinct patterns of muscle activity were measured during the listening and processing of emotional prosody in these two groups. In autistic adults, it could be posited that, during the listening to social stimuli, facial muscles initially tensed up significantly and then relaxed, possibly because of the unpleasant nature of the auditory experience. This finding may align with behavioral results indicating that autistic adults tend to assign unpleasantness ratings to vocal stimuli.^45^ This pattern might also reflect a disengagement or regulatory response, possibly related to the auditory experience; however, as we did not measure affective valence or arousal, these interpretations should be considered with caution.

Another explanation is that the task itself may have placed greater attentional or cognitive demands on autistic participants. According to the STORM model,^44^ motor resonance is not purely reflexive but can be modulated by top-down factors such as task difficulty and social relevance. Increased cognitive effort may therefore reduce the availability of resources for spontaneous mimicry or facial motor resonance. Finally, our results align with previous neuroimaging studies reporting decreased engagement of the somatosensory system during emotion processing in autism,^46^ suggesting broader differences in embodied emotional responses.

The current study indicates a difference in automatic or controlled processes in response to emotional prosody in autism. Indeed, controlled processes, such as choosing the most smiling sound from a pair of similar sounds, appear to be preserved in autistic adults. This type of process relies on low-level acoustic processing, involving a purely frequency analysis that remains intact (or even enhanced) in autism.^47^^,^^48^^,^^49^ The preserved perceptual representations and good recognition of vocal emotions in adults with autism are consistent with a compensatory strategy for controllable behaviors.^50^ The use of acoustic cues similar to those of neurotypical adults may have been acquired through experience and repeated exposure to such information, contrary to the automatic facial response to vocal emotion that was absent in autistic adults.

Interestingly, the similar mental representation of vocal smiles and the equivalent performance in emotional prosody recognition between autistic and neurotypical adults raise questions about impaired recognition observed in several studies.^10^^,^^15^ In the visual modality, it has been suggested^51^ that while the representation of emotions may be similar between autistics and neurotypicals (possibly even more precise for autistics), further differences in recognition and in subsequent response to emotions could be attributed to autistic people being less guided by emotion representation as *a prior* in the Bayesian framework.^52^

Furthermore, the “double empathy” problem posits that autistic and neurotypical individuals may have mutual misunderstandings rather than a deficit on one side,^53^ suggesting a disconnect in reciprocal emotional understanding between autistic and neurotypical individuals. Consistent with this theory, the present study showed that autistic adults exhibited a lack of motor resonance while accurately recognizing emotional prosody, suggesting that their understanding of emotions is different rather than impaired, and that difficulties in reciprocity are not a result of atypical emotional representations. This absence of motor resonance aligns with previous findings indicating challenges in the production and response to facial emotions in individuals with autism.^1^ The absence of congruent motor activity in response to emotional prosody, along with the lack of correlation between accuracy and muscular activity, suggests that emotion embodiment in autism may fundamentally differ from that in neurotypicals. Embodied emotion theory states that emotional experiences are deeply integrated with physical responses, such as facial expressions and bodily sensations. These processes play a crucial role in how emotions are experienced and understood.^54^ Consistent with this theory, it has been proposed that individuals with autism may experience a disconnect or alteration in how emotions are physically felt, expressed, and processed.^55^ For instance, studies indicate that individuals with autism often show atypical facial expressions and reduced facial muscle activity in response to emotional stimuli, suggesting differences in embodied emotional processing.^1^^,^^56^ This suggests that embodied, automatic responses to emotional stimuli, such as smiling or frowning, may be less prevalent or different in individuals with autism. Nevertheless, despite differences in motor resonance, autistic adults were able to recognize emotional prosody accurately, indicating that autistic individuals might use alternative, more cognitive strategies for emotion recognition.^57^

It remains to be explored whether the absence of motor resonance observed here is unique to prosodic cues or whether it reflects a broader difficulty in responding to emotional vocalizations such as laughter or musical expression. Merging evidence indicates that autistic individuals exhibit atypical behavioral and neural responses to social laughter,^30^^,^^31^ and that vocal emotions—even in the absence of visual cues—can elicit facial muscle activity in neurotypical individuals.^35^^,^^36^ Investigating motor resonance in response to such nonverbal vocal stimuli could help determine whether the differences observed in autism reflect a modality-specific atypicality or a broader alteration in embodied emotional processing.

The atypicalities in motor resonance observed in autistic adults may stem from difficulties experienced during childhood. In infancy, emotional resonance is crucial for developing accurate perception and response to emotions, even before the emergence of empathetic abilities.^58^^,^^59^ Such an early deficit in the embodiment of emotion would lead to atypical emotional responses and, subsequently, challenges in social interaction and reciprocity. As previously indicated, emotional resonance is an automatic process; therefore, early difficulties in resonance to vocal emotions are likely to persist and impact emotional reactivity in adulthood.

Interestingly, the absence of motor resonance in the autistic group was not characterized by a global facial hypotonus. On the contrary, autistic adults showed a tonic phase of muscle activation followed by relaxation, regardless of the emotional content of the sentence. This pattern suggests a lack of emotion-specific modulation rather than a general reduction in muscular tone.^60^^,^^61^ In contrast, neurotypical participants showed sustained *Zygomaticus major* activity that was selectively enhanced in response to vocal smiles, indicating a congruent motor response. The contrast between a flat, non-differentiated pattern in the autistic group and a condition-sensitive response in neurotypicals supports the interpretation that emotional embodiment—via motor resonance—is selectively altered in autism.

Nevertheless, the absence of facial EMG activation in the autistic group does not necessarily indicate a lack of emotional experience. Internal arousal in response to vocal emotion may occur without visible motor expression, for example, via autonomic variation. To draw firmer conclusions about embodied responses, future studies should include complementary measures of autonomic nervous system activity.

### Limitations of the study

The relatively small sample size limits the generalizability of our findings, particularly in light of the substantial interindividual variability in emotional prosody processing reported within the autism spectrum.^62^^,^^63^^,^^64^ The results were obtained from verbally autistic adults, the majority of whom did not present with intellectual disability or co-occurring neurodevelopmental or psychiatric conditions. Although there was some variability in cognitive and symptom profiles, the sample did not include minimally verbal individuals. Therefore, caution is warranted in extending these findings to more diverse autistic populations. Similarly, age was not included as a covariate due to the limited sample size and non-normal age distribution in the ASD group. Future studies should aim to recruit larger and more heterogeneous samples to assess the robustness and generalizability of the observed effects. Additionally, incorporating explicit emotional imitation tasks may help clarify whether autistic individuals can voluntarily engage in motor mimicry and how this ability relates to spontaneous facial expressions.^65^ Although participants were not informed of the EMG’s specific purpose and received no instructions regarding facial expression, the possibility of subtle voluntary modulation of facial responses cannot be entirely ruled out.

Finally, adding a spontaneous vocal smile production task would allow for a direct comparison between expressive features, perceptual representations, and associated motor resonance mechanisms. This would be achievable considering the protocol’s relatively short duration (18 min), and given that, as it currently stands, no group differences in performance or engagement were observed, limiting the likelihood that fatigue or motivational factors significantly influenced the results.

This study offers clinical insights; specifically, the finding that the representation of vocal smiles was similar between autistic and neurotypical adults sheds light on the abilities of autistic adults to use the relevant acoustic indices to classify vocal emotion. It would be advantageous to support patients with autism with robust representations of vocal emotions in applying these acquired representations in their everyday lives. This could be accomplished through speech and language therapy or psychomotor rehabilitation, with a focus on the sensory and motor embodiment of emotions. The goal would be to enhance their ability to adapt emotional responses to social contexts by utilizing efficient pre-existing representations and strengthening their understanding and application of these emotions in real-life situations.

The study of embodied emotion in autism reveals important differences in how emotions are integrated and expressed in this population. While autistic individuals may not exhibit typical motor resonance, their ability to understand and recognize emotions suggests that they experience emotions in a way that is different but not necessarily deficient. These findings challenge traditional views of emotional deficits in autism and highlight the need for a more nuanced understanding of emotional processing in this population. Additionally, the present study provides new insights into the lack of synchrony for emotional prosody in autistic adults.

## Resource availability

### Lead contact

Requests for further information and resources should be directed to and will be fulfilled by the lead contact, Marie Gomot (gomot@univ-tours.fr).

### Materials availability

This study did not generate new unique reagents or materials.

### Data and code availability

• All data reported in this article are available from the lead contact upon request. Access will be granted unconditionally to named individuals, in accordance with the conditions of our ethics approval and general ethical procedures governing the reuse of the present data.
• This article does not report the original code.
• Any additional information required to reanalyze the data reported in this article is available from the lead contact upon request.

## STAR★Methods

### Key resources table

**Table:** 

REAGENT or RESOURCE	SOURCE	IDENTIFIER
**Software and algorithms**		

MNE-Python	Gramfort et al.^66^	https://doi.org/10.1016/j.neuroimage.2013.10.027.
RStudio 4.2.2	R Core Team^67^	https://cran.r-project.org/bin/windows/base/old/4.2.2/
CLEESE	Burred et al.^68^	https://doi.org/10.1371/journal.pone.0205943
JASP 0.19.3	JASP Team (2025)	https://jasp-stats.org/previous-versions/

### Experimental model and study participant details

#### Participants

Twenty autistic (ASD) and 21 neurotypical (NT) adults were included in the study. Autistic participants were recruited through the Autism Resource Center of Center Val de Loire and diagnosed by a clinical team using DSM-5 criteria, confirmed with ADOS-2 and ADI-R.^8^^,^^69^ NT participants’ verbal and non-verbal abilities were assessed with selected WAIS-IV subtests,^70^ while ASD participants completed the full scales. All participants completed the Autism Quotient (AQ) and Empathy Quotient (EQ).^71^^,^^72^ NT participants reported no developmental language or sensorimotor difficulties. Both groups had no history of central nervous system disorders, metabolic or infectious diseases, epilepsy, or hearing impairments. Hearing levels were verified through a brief subjective audiometry test. A detailed group description is provided in Table 1.

**Table 1:** Characteristics of neurotypical (NT) and autistic (ASD) group (mean ± standard deviation)

	NT	ASD	Comparison
Sex	7 ♀ 14♂	7 ♀ 13♂	*χ*2 = 0; *p* = 1
Age	26.1 ± 6	31.4 ± 10	t(30) = 2.2; *p* = 0.04
vIQ	125.5 ± 10 (19)	117.6 ± 20 (18)	t(24) = −1.6; *p* = 0.59
nvIQ	106.3 ± 12 (19)	102.7 ± 18 (18)	t(29) = −0.5; *p* = 0.12
AQ	15.8 ± 7 (18)	33.9 ± 7 (18)	t(34) = 7.3; *p* < 0.001
EQ	44.2 ± 9 (18)	24.9 ± 15 (18)	t(30) = −4.8; *p* < 0.001
ADOS	/	10.4 ± 6 (18)	/

vIQ: verbal intelligence quotient calculated from the WAIS-IV in ASD, and composite score calculated from vocabulary and similarities subtests in NT. nvIQ: non-verbal intelligence quotient calculated from the WAIS-IV in ASD, and composite score calculated from matrix reasoning and cubes subtests in NT. ADOS: score calculated as the sum of the subdimensions of communication and social interaction behaviors. The values in brackets correspond to the number of values included in the calculation of the mean and standard deviation due to missing data.

#### Ethics statement

The study was approved by *Comité de Protection des Personnes* CPP Est I (PROSCEA2017/23; ID RCB: 2017-A00756-47). All methods in this study were carried out in accordance with the relevant guidelines and regulations, and all data in this study were obtained with informed consent from all subjects and/or their legal guardian(s).

### Method details

#### Preliminary validation of vocal smile model – Reverse correlation

Mental representations of vocal smiles were assessed in both groups using the method and stimuli from Ponsot et al.^29^ A French male speaker’s/a/phoneme with constant pitch was recorded, and a 500 ms stationary segment with constant spectral energy was selected. Spectral variants were generated by modulating the original sound using a random frequency equalizer. This equalizer, based on 25 log-spaced frequency points (100–10,000 Hz), interpolated linearly between gain values (in dB) drawn from Gaussian distributions (SD = 5 dB, clipped at ±2.5 SD) using the Cleese Python toolbox.^68^

#### Main task

The task from Merchie et al.^34^ was presented using semantically neutral sentences artificially modified to have Smiling and Unsmiling prosody based on the model of vocal smile from Ponsot et al.^29^ Vocal smile was characterized as a robust internal representation in adults, based on the vocal sound acoustic features: compared to neutral voice, smile was associated with an upward shift in the frequencies of the first and second formants (F1 and F2) and an increase in the energy of the second, third, and fourth formants (F2, F3, and F4). The sentences used were filtered based on these acoustic characteristics, as in Arias et al.^32^^,^^33^ and Merchie et al.^34^ Ten different speakers (five male and five female) produced the sentences.

**Figure 5:**
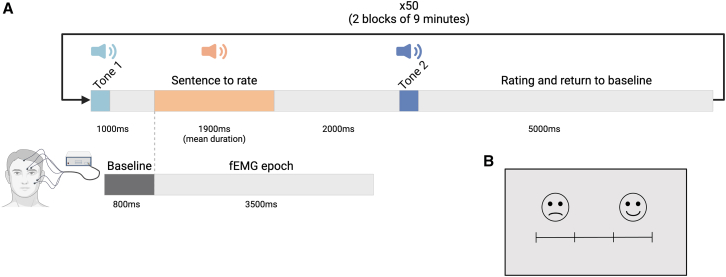
Experimental design and response mapping in the EMG task (A) Timeline of a trial in the experimental sequence. Tone 1 indicated the beginning of a new trial, tone 2 indicated the time when the subject should rate the sentence. (B) Rating scale displayed on screen throughout the duration of the task; fEMG: facial ElectroMyoGraphy. Adapted from Merchie et al.^34^

Two 300 ms tones (440 Hz, 275 Hz) were generated using Praat^73^ with energy normalization and fade-in/fade-out. The first tone signaled trial onset, followed by a sentence after 1000 ms. A 2000 ms silence preceded the second tone, marking the start of the 5000 ms rating period. Trial structure and response mapping are illustrated in Figure 5A.

Participants were seated comfortably 70 cm from the screen (1980x1080 px) and speakers. The sound intensity was controlled and maintained between 58- and 62-dB SPL.

The following instruction was given to participants: ‘You will hear sentences, and you will be asked to rate how joyfully or unjoyfully the sentence was pronounced. You should not judge the content of the sentence but the way it was pronounced’. For the behavioral data associated to facial EMG and pupil recording, judgements were recorded through a 4-key response pad. To minimize the risk of voluntary control over facial muscle activity, the purpose of the EMG electrodes was not disclosed to participants, and no specific instructions were given regarding facial expression. The displayed image represented the rating scale: the two right keys corresponded to two levels of Smile intensity and the two left keys corresponded to two levels of Unsmile intensity (Figure 5B).

#### fEMG recording

Facial EMG (fEMG) was recorded using 4 mm Ag-AgCl electrodes (EL254 and EL254S) with the BIOPAC MP36 system and AcqKnowledge 4.1 software (BIOPAC Systems Inc., Goleta, CA). To measure the *Zygomaticus major* (ZM) and *Corrugator supercilii* (CS) muscles, electrodes were placed on the participant’s left face and a reference electrode was placed on the middle of the forehead at the hairline. The skin was cleaned with an abrasive paste to remove traces of moisturizer, makeup, or sweat, following Fridlund and Cacioppo.^74^ Data were recorded at a sampling rate of 1024 Hz.

Participants were informed that facial electrodes would be used to record subtle muscle activity, but no specific information was provided about the purpose of the EMG recording or its relation to emotional expression.

### Quantification and statistical analysis

#### Pre-processing and measurements

##### Reverse correlation model analysis

A model extraction was computed for each participant as the gain difference between the chosen as smiling and as unsmiling for each frequency, referred as spectral modifications. Then, to compare group’s model of vocal smile, a statistical analysis using a t-test was conducted on the spectral modifications at each modulated frequency.

To evaluate if the recognition of applied filters was related with the representation of vocal smile. The mean Euclidian distance for each frequency between the combined model of ASD and NT and a participant’s model was computed as the Kernel Distance.

##### Behavioral measures in the main task: perceptual accuracy

In the following parts, the acoustical modifications of the sentences are referred to as Filter (Smiling-F vs. Unsmiling-F) and the ratings of the smiliness of the sentences are referred to as Choice (Smile-C vs. Unsmile-C). Choice is based on participants' trial-by-trial perceptual judgments. Specifically, each sentence was categorized post hoc according to whether the participant labeled it as ‘*Smile’* or ‘*Unsmile’*. This allowed us to test whether facial muscle responses aligned with participants’ subjective perception of emotional prosody, independently of the acoustic manipulation. Note that Choice is not an experimental manipulation but a perceptual variable derived from participants' responses.

Perceptual accuracy was calculated as the number of Correct responses divided by the total number of Smiling-F and Unsmiling-F filtered sentences (80 for each participant) to reflect the accurate rating of both filters (Equation 1). Accuracy was compared to the chance level (50%) to evaluate the recognition of emotional prosodic modulation.

(Equation 1) $$Perceptual\;accuracy=\frac{nSmilin{g-F}_{smile-C}+nUnsmilin{g-F}_{unsmile-C}}{nSmiling-F+nUnsmiling-F}$$

*nSmiling-F*_*smile-C*_*: number of trials for smiling filtered sentences rated as smile; nUnsmiling-F*_*unsmile-C*_*: number of trials for Unsmiling filtered sentences rated as unsmile; nSmiling-F: total of trials for the Smiling Filter; nUnsmiling-F: total of trials for the Unsmiling Filter.*

##### fEMG processing

fEMG data were analyzed and pre-processed using the MNE toolbox^66^ in Python 3.9.4. The data were filtered with a 50 Hz IIR high-pass filter and a 250 Hz low-pass filter. Significant movements artifacts were manually removed. The data were then segmented into epochs with an 800 ms pre-stimulus baseline and a 3500 ms duration, starting at the sentence onset. The absolute value of the muscular activity was smoothed and averaged with a 300 ms sliding window, then normalized using z-scores based on the baseline of the current trial. On average, 22 ± 17% (mean ± SD) of trials were removed in autistic, and 20 ± 11% in neurotypical adults due to excessive movement artifacts. None of the participants were excluded from this study due to missing or poor-quality data.

##### Statistical analysis

Statistical analyses were performed using Rstudio 4.2.2^67^^,^^75^ with the ggplot2,^76^ ez,^77^ tidyverse,^78^ and dplyr^79^ packages.

Pearson’s Correlations between the Kernel Distance and the Accuracy and between the Accuracy and the AQ/EQ scores were computed.

To evaluate the effect of listening sentences on muscular tonus in both groups the mean muscular activity was computed during Listening (0–1900 ms) and during Processing (1900–4000 ms) of sentences. A repeated-measures ANOVA on mean muscular activity with Timing (Listening vs. Processing) and Muscle (ZM vs. CS) as within factor, and Group (ASD vs. NT) as between subject factors. For each muscle activity, the effects of Filter and Choice were estimated through the mean amplitude within selected time windows, both between and within groups (ASD and NT). To specify the significant effect in the selected time window, a repeated-measures ANOVA (Filter x Choice) was performed on mean muscular activity. ANOVAs were focused on a chosen 500 ms window (2900–3400 ms) during this phase in accordance with previous study, which identified this time window as showing a significant increase in z-normalized fEMG activity in response to vocal smiles.^34^

In case of significant main effect or interaction, post-hoc Student’s tests were performed with a Bonferroni’s correction for multiple comparisons.

To assess group differences in case of no significant difference in the frequentist approach, we conducted a Bayesian statistical analysis on JASP (Version 0.19.3).

## Acknowledgments

We thank all volunteers for their time and participation in this study. We would also like to thank Shasha Morel-Kohlmeyer and Marine Bessé for helping with the recruitment of participants. Annabelle Merchie was funded by the FEANS (Fondation Européenne pour l’Avancement des Neurosciences) and the ANR (Agence Nationale de la Recherche, ANR-19-CE37-0022-01). The funding sources were not involved in the study design, data collection, analysis, or the writing of the report.

Please contact the corresponding author for data and experimental stimuli requests.

## Author contributions

A.M., N.A-H., C.W., J-J.A., and M.G. designed the study. A.M. and Z.R. performed data acquisition. A.M., J-J.A., and M.G. were responsible for data and statistical analyses. E. H-D. and F. B-B. were responsible for the patients’ inclusion. A.M. and M.G. wrote the first version of the article. All authors were involved in preparing and reviewing the article.

## Declaration of interests

All authors declare that they have no conflicts of interest.

## References

[1] Trevisan D.A., Hoskyn M., Birmingham E. (2018). Facial Expression Production in Autism: A Meta-Analysis. Autism Res..

[2] McIntosh D.N., Reichmann-Decker A., Winkielman P., Wilbarger J.L. (2006). When the social mirror breaks: deficits in automatic, but not voluntary, mimicry of emotional facial expressions in autism. Dev. Sci..

[3] Yirmiya N., Sigman M.D., Kasari C., Mundy P. (1992). Empathy and Cognition in High-Functioning Children with Autism. Child Dev..

[4] Faso D.J., Sasson N.J., Pinkham A.E. (2015). Evaluating posed and evoked facial expressions of emotion from adults with autism spectrum disorder. J. Autism Dev. Disord..

[5] Grossman R.B. (2015). Judgments of social awkwardness from brief exposure to children with and without high-functioning autism. Autism.

[6] Paul R., Augustyn A., Klin A., Volkmar F.R. (2005). Perception and Production of Prosody by Speakers with Autism Spectrum Disorders. J. Autism Dev. Disord..

[7] Shriberg L.D., Paul R., McSweeny J.L., Klin A.M., Cohen D.J., Volkmar F.R. (2001). Speech and prosody characteristics of adolescents and adults with high-functioning autism and Asperger syndrome. J. Speech Lang. Hear. Res..

[8] Lord C., Risi S., Lambrecht L., Cook E.H., Leventhal B.L., DiLavore P.C., Pickles A., Rutter M. (2000). The Autism Diagnostic Observation Schedule—Generic: A Standard Measure of Social and Communication Deficits Associated with the Spectrum of Autism. J. Autism Dev. Disord..

[9] Wang J.-E., Tsao F.-M. (2015). Emotional prosody perception and its association with pragmatic language in school-aged children with high-function autism. Res. Dev. Disabil..

[10] Hubbard D.J., Faso D.J., Assmann P.F., Sasson N.J. (2017). Production and perception of emotional prosody by adults with autism spectrum disorder: Affective prosody in ASD. Autism Res..

[11] Rosenblau G., Kliemann D., Dziobek I., Heekeren H.R. (2017). Emotional prosody processing in autism spectrum disorder. Soc. Cogn. Affect. Neurosci..

[12] Zhang M., Xu S., Chen Y., Lin Y., Ding H., Zhang Y. (2022). Recognition of affective prosody in autism spectrum conditions: A systematic review and meta-analysis. Autism.

[13] Golan O., Baron-Cohen S., Hill J.J., Rutherford M.D. (2007). The ‘Reading the Mind in the Voice’ Test-Revised: A Study of Complex Emotion Recognition in Adults with and Without Autism Spectrum Conditions. J. Autism Dev. Disord..

[14] Peppé S., McCann J., Gibbon F., O’Hare A., Rutherford M. (2007). Receptive and Expressive Prosodic Ability in Children With High-Functioning Autism. J. Speech Lang. Hear. Res..

[15] Leung F.Y.N., Sin J., Dawson C., Ong J.H., Zhao C., Veić A., Liu F. (2022). Emotion recognition across visual and auditory modalities in autism spectrum disorder: A systematic review and meta-analysis. Dev. Rev..

[16] Brewer R., Biotti F., Catmur C., Press C., Happé F., Cook R., Bird G. (2016). Can Neurotypical Individuals Read Autistic Facial Expressions? Atypical Production of Emotional Facial Expressions in Autism Spectrum Disorders. Autism Res..

[17] Merchie A., Ranty Z., Adl Zarrabi A., Bonnet-Brilhault F., Houy-Durand E., Aucouturier J.-J., Gomot M. (2025). Intact representation of vocal smile in autism: A reverse correlation approach. Res. Autism.

[18] Helt M.S., Eigsti I.-M., Snyder P.J., Fein D.A. (2010). Contagious Yawning in Autistic and Typical Development: Contagious Yawning. Child Dev..

[19] Helt M.S., Sorensen T.M., Scheub R.J., Nakhle M.B., Luddy A.C. (2021). Patterns of Contagious Yawning and Itching Differ Amongst Adults With Autistic Traits vs. Psychopathic Traits. Front. Psychol..

[20] Senju A., Maeda M., Kikuchi Y., Hasegawa T., Tojo Y., Osanai H. (2007). Absence of contagious yawning in children with autism spectrum disorder. Biol. Lett..

[21] Beall P.M., Moody E.J., McIntosh D.N., Hepburn S.L., Reed C.L. (2008). Rapid facial reactions to emotional facial expressions in typically developing children and children with autism spectrum disorder. J. Exp. Child Psychol..

[22] Magnée M.J.C.M., De Gelder B., Van Engeland H., Kemner C. (2007). Facial electromyographic responses to emotional information from faces and voices in individuals with pervasive developmental disorder. J. Child Psychol. Psychiatry.

[23] Oberman L.M., Winkielman P., Ramachandran V.S. (2009). Slow echo: facial EMG evidence for the delay of spontaneous, but not voluntary, emotional mimicry in children with autism spectrum disorders. Dev. Sci..

[24] Norscia I., Zanoli A., Gamba M., Palagi E. (2020). Auditory Contagious Yawning Is Highest Between Friends and Family Members: Support to the Emotional Bias Hypothesis. Front. Psychol..

[25] Giganti F., Esposito Ziello M. (2009). Contagious and spontaneous yawning in autistic and typically developing children. Curr. Psychol. Lett. Behav. Brain Cogn.

[26] Rychlowska M., Jack R.E., Garrod O.G.B., Schyns P.G., Martin J.D., Niedenthal P.M. (2017). Functional Smiles: Tools for Love, Sympathy, and War. Psychol. Sci..

[27] Dawson G., Hill D., Spencer A., Galpert L., Watson L. (1990). Affective exchanges between young autistic children and their mothers. J. Abnorm. Child Psychol..

[28] Niedenthal P.M., Mermillod M., Maringer M., Hess U. (2010). The Simulation of Smiles (SIMS) model: Embodied simulation and the meaning of facial expression. Behav. Brain Sci..

[29] Ponsot E., Arias P., Aucouturier J.-J. (2018). Uncovering mental representations of smiled speech using reverse correlation. J. Acoust. Soc. Am..

[30] Cai C.Q., Lavan N., Chen S.H.Y., Wang C.Z.X., Ozturk O.C., Chiu R.M.Y., Gilbert S.J., White S.J., Scott S.K. (2024). Mapping the differential impact of spontaneous and conversational laughter on brain and mind: an fMRI study in autism. Cereb. Cortex.

[31] Cai C.Q., White S.J., Chen S.H.Y., Mueller M.A.E., Scott S.K. (2024). Autistic adults perceive and experience laughter differently to non-autistic adults. Sci. Rep..

[32] Arias P., Belin P., Aucouturier J.-J. (2018). Auditory smiles trigger unconscious facial imitation. Curr. Biol..

[33] Arias P., Bellmann C., Aucouturier J.-J. (2021). Facial mimicry in the congenitally blind. Curr. Biol..

[34] Merchie A., Ranty Z., Aguillon-Hernandez N., Aucouturier J.-J., Wardak C., Gomot M. (2024). Emotional contagion to vocal smile revealed by combined pupil reactivity and motor resonance. Sci. Rep..

[35] Lima C.F., Arriaga P., Anikin A., Pires A.R., Frade S., Neves L., Scott S.K. (2021). Authentic and posed emotional vocalizations trigger distinct facial responses. Cortex.

[36] Vilaverde R.F., Horchak O.V., Pinheiro A.P., Scott S.K., Korb S., Lima C.F. (2024). Inhibiting orofacial mimicry affects authenticity perception in vocal emotions. Emotion.

[37] Lindström R., Lepistö-Paisley T., Makkonen T., Reinvall O., Nieminen-von Wendt T., Alén R., Kujala T. (2018). Atypical perceptual and neural processing of emotional prosodic changes in children with autism spectrum disorders. Clin. Neurophysiol..

[38] Dimberg U., Thunberg M., Elmehed K. (2000). Unconscious Facial Reactions to Emotional Facial Expressions. Psychol. Sci..

[39] Hess U., Blairy S. (2001). Facial mimicry and emotional contagion to dynamic emotional facial expressions and their influence on decoding accuracy. Int. J. Psychophysiol..

[40] Olszanowski M., Wróbel M., Hess U. (2020). Mimicking and sharing emotions: a re-examination of the link between facial mimicry and emotional contagion. Cogn. Emot..

[41] Dimberg U. (1982). Facial Reactions to Facial Expressions. Psychophysiology.

[42] Sonnby-Borgström M. (2002). Automatic mimicry reactions as related to differences in emotional empathy. Scand. J. Psychol..

[43] Korb S., With S., Niedenthal P., Kaiser S., Grandjean D. (2014). The Perception and Mimicry of Facial Movements Predict Judgments of Smile Authenticity. PLoS One.

[44] Wang Y., Hamilton A.F.D.C. (2012). Social top-down response modulation (STORM): a model of the control of mimicry in social interaction. Front. Hum. Neurosci..

[45] Michel L., Ricou C., Bonnet-Brilhault F., Houy-Durand E., Latinus M. (2024). Sounds Pleasantness Ratings in Autism: Interaction Between Social Information and Acoustical Noise Level. J. Autism Dev. Disord..

[46] Fanghella M., Gaigg S.B., Candidi M., Forster B., Calvo-Merino B. (2022). Somatosensory evoked potentials reveal reduced embodiment of emotions in autism. J. Neurosci..

[47] Bonnel A., Mottron L., Peretz I., Trudel M., Gallun E., Bonnel A.-M. (2003). Enhanced pitch sensitivity in individuals with autism: a signal detection analysis. J. Cogn. Neurosci..

[48] Bonnel A., McAdams S., Smith B., Berthiaume C., Bertone A., Ciocca V., Burack J.A., Mottron L. (2010). Enhanced pure-tone pitch discrimination among persons with autism but not Asperger syndrome. Neuropsychologia.

[49] Samson F., Hyde K.L., Bertone A., Soulières I., Mendrek A., Ahad P., Mottron L., Zeffiro T.A. (2011). Atypical processing of auditory temporal complexity in autistics. Neuropsychologia.

[50] Livingston L.A., Shah P., Happé F. (2019). Compensatory strategies below the behavioural surface in autism: a qualitative study. Psychiatry.

[51] Keating C.T., Ichijo E., Cook J.L. (2023). Autistic adults exhibit highly precise representations of others’ emotions but a reduced influence of emotion representations on emotion recognition accuracy. Sci. Rep..

[52] Lawson R.P., Rees G., Friston K.J. (2014). An aberrant precision account of autism. Front. Hum. Neurosci..

[53] Milton D.E.M. (2012). On the ontological status of autism: the ‘double empathy problem’. Disabil. Soc..

[54] Niedenthal P.M. (2007). Embodying Emotion. Science.

[55] Eigsti I.-M. (2013). A Review of Embodiment in Autism Spectrum Disorders. Front. Psychol..

[56] Sato W., Fujimura T., Kochiyama T., Suzuki N. (2013). Relationships among Facial Mimicry, Emotional Experience, and Emotion Recognition. PLoS One.

[57] Jones C.R.G., Pickles A., Falcaro M., Marsden A.J.S., Happé F., Scott S.K., Sauter D., Tregay J., Phillips R.J., Baird G. (2011). A multimodal approach to emotion recognition ability in autism spectrum disorders. J. Child Psychol. Psychiatry.

[58] Meltzoff A.N., Decety J. (2003). What imitation tells us about social cognition: a rapprochement between developmental psychology and cognitive neuroscience. Philos. Trans. R. Soc. Lond. B Biol. Sci..

[59] de Waal F.B.M., Ferrari P.F. (2010). Towards a bottom-up perspective on animal and human cognition. Trends Cogn. Sci..

[60] Al Abdulmohsen T., Kruger T.H.C. (2011). The contribution of muscular and auditory pathologies to the symptomatology of autism. Med. Hypotheses.

[61] Serdarevic F., Ghassabian A., van Batenburg-Eddes T., White T., Blanken L.M.E., Jaddoe V.W.V., Verhulst F.C., Tiemeier H. (2017). Infant muscle tone and childhood autistic traits: A longitudinal study in the general population. Autism Res..

[62] Grossman R.B., Edelson L.R., Tager-Flusberg H. (2013). Emotional facial and vocal expressions during story retelling by children and adolescents with high-functioning autism. J. Speech Lang. Hear. Res..

[63] Rutherford M.D., Baron-Cohen S., Wheelwright S. (2002). Reading the Mind in the Voice: A Study with Normal Adults and Adults with Asperger Syndrome and High Functioning Autism. J. Autism Dev. Disord..

[64] Paul R., Orlovski S.M., Marcinko H.C., Volkmar F. (2009). Conversational behaviors in youth with high-functioning ASD and Asperger syndrome. J. Autism Dev. Disord..

[65] Heaton P., Williams K., Cummins O., Happé F.G.E. (2007). Beyond perception: musical representation and on-line processing in autism. J. Autism Dev. Disord..

[66] Gramfort A., Luessi M., Larson E., Engemann D.A., Strohmeier D., Brodbeck C., Parkkonen L., Hämäläinen M.S. (2014). MNE software for processing MEG and EEG data. Neuroimage.

[67] R Core Team (2022).

[68] Burred J.J., Ponsot E., Goupil L., Liuni M., Aucouturier J.-J. (2019). CLEESE: An open-source audio-transformation toolbox for data-driven experiments in speech and music cognition. PLoS One.

[69] Lord C., Rutter M., Le Couteur A. (1994). Autism Diagnostic Interview-Revised: A revised version of a diagnostic interview for caregivers of individuals with possible pervasive developmental disorders. J. Autism Dev. Disord..

[70] Wechsler D. (2012).

[71] Baron-Cohen S., Wheelwright S., Skinner R., Martin J., Clubley E. (2001). The autism-spectrum quotient (AQ): evidence from Asperger syndrome/high-functioning autism, males and females, scientists and mathematicians. J. Autism Dev. Disord..

[72] Baron-Cohen S., Wheelwright S. (2004). The Empathy Quotient: An Investigation of Adults with Asperger Syndrome or High Functioning Autism, and Normal Sex Differences. J. Autism Dev. Disord..

[73] Boersma P. (2002). Praat, a system for doing phonetics by computer. Glot Int..

[74] Fridlund A.J., Cacioppo J.T. (1986). Guidelines for human electromyographic research. Psychophysiology.

[75] R Core Team (2021).

[76] Wickham H. (2016).

[77] Lawrence M.A. (2016). ez: Easy Analysis and Visualization of Factorial Experiments. http://github.com/mike-lawrence/ez.

[78] Wickham H., Averick M., Bryan J., Chang W., McGowan L., François R., Grolemund G., Hayes A., Henry L., Hester J. (2019). Welcome to the Tidyverse. J. Open Source Softw..

[79] Wickham H., François R., Henry L., Müller K. (2021). dplyr: A Grammar of Data Manipulation. https://CRAN.R-project.org/package=dplyr.

